# Chicago Medical Society

**Published:** 1869-09

**Authors:** 


### CHICAGO MEDICAL SOCIETY.

FRIDAY EVENING, JULY 23, 1869.

The regular semi-monthly meeting of this Society was called to order, President Bogue in the Chair.

The Secretary read the minutes of the last meeting, which were approved and ordered to be placed on file.

The next order of business was the proposals for membership.

The name of Dr. F. W. Calkins was proposed by Drs. Quales and Parks.

Dr. Quales then proceeded to read his report on the Sanitary Condition of the North Division for the quarter ending June 30, 1869. The Doctor gave a detailed account of the meteorological observations he had taken, and said that from the unusual amount of rain during May and June, and the consequent dampness of the atmosphere, it would be natural to infer that they had a deleterious influence upon the sanitary condition in general, and more especially in such localities where the drainage is insufficient. This was confirmed by practical observation. He stated that it had been his experience under these circumstances that diarrhœa, dysentery, etc., especially among children, manifested themselves earlier, and in a more malignant form. This had been the case during the two preceding years. Fevers, especially intermittent, remittent, and typho-malarial, also rheumatic and neuralgic diseases. Of contagious diseases, there had been 40 cases of small-pox, only 2 of which proved fatal, which was evidence it was of a mild type; measles, 117 cases, 92 occurring among emigrants.

The report of Dr. Quales, upon motion, was accepted and ordered to be placed on file.

Dr. Reid remarked that he had met with quite a number of cases of variola, all of which were quite mild; also a number of cases of measles and scarlatina, and within the past two weeks a considerable number of cases of infantile diarrhœa had been met with. Upon being asked what treatment he generally pursued in the latter disease, the Doctor said he usually made the following prescription:—

℞. Calomel........................gr. ij.

Morph. sulph....................gr. $\frac{1}{10}$—$\frac{1}{12}$

Sugar............................gr. xij.

Ft. chart No. xij. One powder to be given every two or three hours, according to circumstances. Also sometimes employed hydr. cum creta, sub. nit. bismuth and rub. villosus.

Reported one case of a woman who had profuse rice water discharges, and had Asiatic cholera been prevalent, should have decided to be such, but under the circumstances could call it nothing but a sporadic case.

Dr. Marguerat remarked that he had seen large doses of brandy prove beneficial in the treatment of cholera infantum; also pepsin, in $\frac{1}{2}$ gr. doses every two hours. Thinks the practitioner should not trust the nurse to administer morphine to children.

Dr. Fitch says he has been in the habit of using morphine in doses ranging from $\frac{1}{60}$ to $\frac{1}{120}$ gr. in the treatment of cholera infantum, for a number of years, but always weighs it and puts it up himself when for children. He stated that the last case but one he had prescribed for that evening, was one of cholera infantum. Gave morphine, gr. $\frac{1}{100}$; bismuth and sugar, each, gr. ij. at a dose. The child was teething, and he scarified the gums, which, he says, he invariably does under such circumstances. Also applied a sinapism to epigastrium sufficiently long at a time to keep up a redness. Always advises giving the powder dry on the tongue, and as little water as possible. If the milk curdles on the child's stomach, he usually substitutes arrowroot, or something of the kind.

Dr. Paoli wished to know if opium is a proper remedy to be generally employed in the treatment of cholera infantum. He is of the opinion that we do little in giving this drug to assist nature in assimilating the food. He thinks, however, that there are a few cases where it may be beneficially employed, withdrawing the medicine when the secretions become diminished. The Doctor says he usually gives a little calomel combined with the opium, and applies cold water to the head. He does not think there is much nutriment in arrowroot, and always prefers the mother's milk. The tonic treatment, such as columbo and cascarilla, together with sits baths, and warm flannel clothing to keep up the bodily secretion, is the treatment most usually employed by him.

Dr. Fitch thinks that Dr. Paoli does not discriminate between cholera infantum and diarrhœa. There is hyperœmia in these cases in the intestines and not in the brain, and in cases where nothirg can be retained on the stomach the effect of the morphine is to stimulate the brain.

Dr. Paoli says he has never had any success in the treatment of cholera infantum by morphine.

Dr. Parks reported the successful treatment of a case of cholera infantum by the use of pepsin, where there was no digestion.

Dr. Wanzer also reported several cases successfully treated by pepsin combined with $\frac{1}{32}$ of gr. of morphia. The kind employed was Houghton's pepsin, in 2 gr. doses.

Dr. Macdonald has employed the following treatment of cholera infantum, with success:—

℞. Pulverized Charcoal-------------------gr. j.
Sub. Nit. Bismuth -------------------gr. ij.

One powder to be given every two hours; also applied flannel over the bowels.

Dr. Quales says he has seen four cases of cholera infantum, one of which died. He used the pepsin, but is not very favorably impressed with its efficacy.

Dr. Wanzer says when the milk cannot be retained on the child's stomach, he usually gives calomel, gr. $\frac{1}{8}$, with a grain of sugar. Whatever medicine he gives he uses as little water as possible. Port wine and ice has been employed with benefit by him.

Dr. Bogue says he has given the sulphate of morphine in the treatment of cholera infantum, in doses varying from the $\frac{1}{80}$ to the $\frac{1}{100}$ of a grain, for several years, with decided satisfaction.

Dr. Avery says he is in the habit of giving hydr. cum creta, gr. ij.; camph. Doveri, gr. ij., three times a day, sometimes employing an emulsion consisting of—

℞. Al. Ricini------------------------ʒ vj.

Tinct. Opii------------------------gtt. xvij.

Gum Aracia }

Sach. Alba } āā------------------ʒ ij.

Aquæ q. s.------------------------℥ iv.

M. Sig. To be given in teaspoonful doses, pro re nata.

Dr. Fitch is of the opinion that hydrarg. cum creta produces vomiting, and gives calomel combined with bismuth, or creta preparata instead.

The Society then proceeded to miscellaneous business. Subject for discussion at next regular meeting, "The Pathology and Treatment of Chlorosis."

Society adjourned.

